# Seroepidemiology study of Cytomegalovirus and Rubella in pregnant women in Luanda, Angola: geospatial distribution and its association with socio-demographic and clinical-obstetric determinants

**DOI:** 10.1186/s12879-022-07087-x

**Published:** 2022-02-05

**Authors:** Amélia Vueba, Clarissa Faria, Ricardo Almendra, Paula Santana, Maria do Céu Sousa

**Affiliations:** 1 Faculty of Pharmacy, University of Coimbra (FFUC), 3000-548 Coimbra, Portugal; 2 Center for Neuroscience and Cell Biology (CNC), University of Coimbra, Coimbra, Portugal; 3 Centre of Studies on Geography and Spatial Planning (CEGOT), Department of Geography and Tourism, University of Coimbra, Coimbra, Portugal; 4 CEGOT-Centre of Studies in Geography and Spatial Planning and Department of Geography and Tourism, Faculty of Arts and Humanities, University of Coimbra, Coimbra, Portugal

**Keywords:** Anti-Rubella antibodies, Anti-CMV antibodies, Seroprevalence, Rubella infection, Cytomegalovirus infection, Hepatitis B, HIV, Pregnancy, Miscarriages, Spontaneous abortion, Vaccination

## Abstract

**Background:**

Both CMV and Rubella virus infections are associated with the risk of vertical transmission, fetal death or congenital malformations. In Angola, there are no reports of CMV and Rubella studies. Therefore, our objectives were to study the seroprevalence of anti-CMV and anti-Rubella antibodies in pregnant women of Luanda (Angola), identify the risk of primary infection during pregnancy and evaluate the socio-demographic risk factors associated with both infections.

**Methods:**

A prospective cross-sectional study was conducted from August 2016 to May 2017. Specific anti-CMV and anti-Rubella antibodies were quantified by electrochemiluminescence and demographic and clinical data were collected using standardized questionnaire. Bivariate and multivariate logistic regression analysis were used to quantify the effect of clinical and obstetric risk factors on virus seroprevalence.

**Results:**

We recruited 396 pregnant women aged from 15 to 47. Among them, 335 (84.6%) were immune to both CMV and Rubella virus infections, while 8 (2.0%) had active CMV infection and 4 (1.0%) active RV infection but none had an active dual infection. Five women (1.2%) were susceptible to only CMV infection, 43 (10.9%) to only RV infection, and 1 (0.3) to both infections. Multivariate analysis showed a significant association between Rubella virus infection and number of previous births and suffering spontaneous abortion.

**Conclusions:**

Overall, this study showed that there is a high prevalence of anti-CMV and anti-Rubella antibodies in pregnant women in Luanda. It also showed that a small but important proportion of pregnant women, about 11%, are at risk of primary infection with rubella during pregnancy. This emphasizes the need for vaccination.

**Supplementary Information:**

The online version contains supplementary material available at 10.1186/s12879-022-07087-x.

## Background

Cytomegalovirus (CMV) and Rubella virus (RV) infections can be transmitted to the fetus during pregnancy, causing spontaneous abortion, fetal death or congenital malformation [1]. Therefore, early antenatal surveillance is important for the prevention of vertical transmission of these. However, the Angolan National Health Development Plan (2012–2025) does not include the control of diseases such as Rubella and Cytomegalovirus [2]. The absence of a program for early diagnosis and treatment of maternal infections can considerably increase the rates of perinatal morbidity and mortality.

Man is the only reservoir for CMV and the virus transmission occurs by direct or indirect contact with saliva, oropharyngeal secretions, endocervical secretions, urine, sperm, breast milk, tears, blood products or organs, and by vertical transmission [3, 4]. Like other members of the Herpesviridae family, CMV can arrest in the body (latent phase) after a primary infection. In addition, acquired immunity is not completely protective and secondary infections may occur during the latent phase [5]. Vertical transmission rate varies with the type of maternal infection, and is between 30 and 50% during primary infections and 0.1 and 3% during secondary infections (re-infections or reactivations of a latent infection) [3, 5]. The rate is also related to gestational age: 36% in the first trimester, 44% in the second and 77.6% in the last trimester. However, the consequences of CMV infection are more severe when maternal infection occurs before 20 weeks [3, 5].

The most common form of Rubella transmission is through direct contact with respiratory droplets from infected persons [6]. Maternal infection with the rubella virus during the first trimester is often associated with fetal death, miscarriage or adverse neonatal outcome, including heart problems, cataracts and deafness, known as congenital rubella syndrome (CRS), which has a neonatal morbidity [7]. This severity of fetal infection is related to the period of organogenesis, due to the high virus tropism for fetal tissues [8]. Some defects have also been reported from in second trimester [7]. In order to avoid vertical transmission, seronegative women should be detected during the preconception period and their pregnancies planned after immunization [9].

In Angola, there are no reports of CMV and Rubella studies. Therefore, the objectives of this work were to study the prevalence of anti-CMV and anti-Rubella antibodies in pregnant women attending Lucrécia Paim Maternity Hospital (LPMH) of Luanda and to provide a detailed analysis of the geographical distribution. In addition, we aimed to evaluate the risk factors associated with CMV and Rubella infections.

## Methods

### Ethical considerations

The study was approved by the Research Ethics Committee of LPMH through the National Institute of Public Health of the Republic of Angola (No. 301019) (Additional file 1).

Participants gave written informed consent prior to sample collection and for participants younger than 18 years, written informed consent was provided by parents or guardians after a detailed explanation of the objectives of the work.

### Study population

The study population constituted pregnant women undergoing routine antenatal assessment at LPMH, a reference maternity in Luanda, Angola. The women came from all over the country, although mostly from Luanda, the capital of Angola.

The cross-sectional study was conducted from August 2016 to May 2017 and a total of 396 pregnant women, aged from 15 to 47 years, were included in the survey. The pregnancy was confirmed by ultrasonography and laboratory tests, a qualitative rapid immunochromatographic test followed by a quantitative electrochemiluminescence immunoassay of hCG plus the β-hCG subunit in serum (Elecsys hCG + β test). For the obstetric follow-up we counted on the collaboration of the medical and nursing team of the department of obstetrics of the LPMH.

### Sociodemographic, clinical and housing characteristics of the pregnant women

A standardized questionnaire administered on face-to-face interview was used to obtain information on socio-demographic, clinical, and housing characteristics from the women (Additional file 2). The questionnaire was written in Portuguese, the official language in the Republic of Angola.

### Blood sample collection and laboratory procedures

Blood samples were collected and serum samples obtained after centrifugation. The serum samples were immediately transferred (packaged in dry ice) to the Clinical Pathology Service of Clínica Sagrada Esperança (Luanda) and kept at − 80 °C until serological analysis.

The quantification of anti-CMV and anti-Rubella IgG and IgM antibodies was done by Electrochemiluminescence (ECL) using commercially available kits for COBAS e411 (Roche, Sistemas de Diagnósticos Lda) (Batch numbers: 26842601 for CMV IgM; 21219603 for CMV IgG; 23290201 for Rubella IgM; and 26742101 for Rubella IgG), according to the guidelines of the manufacturer.

All antibody levels were expressed as IU/mL and negative versus positive COI (cut-off index) of antibody levels set as follows: anti-CMV IgG < 0.5 vs. > 1.0 IU/mL; anti-CMV IgM < 0.7 vs. > 1.0 IU/mL; ant-RV IgG < 10.0 vs. > 10.0 IU/mL; and anti-RV IgM < 0.8 vs. > 1.0 IU/mL.

Based on the profile of antibodies, the status of the women was classified into four categories: “Previous infection or Immune” [IgG(+)/IgM(−)], “Active (Primary/Latent) infection [IgG(+)/IgM(+)], “Susceptible to primary infection” [IgG(−)/IgM(−)], and “Recent infection” [IgG(−)/IgM(+)].

### Geospatial analysis

The residential addresses of the pregnant women collected during interview were converted into geographic coordinates (latitude and longitude) through the www.google.pt/maps/. The spatial distribution was then assessed through a Kernel Density Function that allowed the estimation of the intensity of events across a surface. The bivariate version of Ripley’s K-function was used to characterize the patterns presented, if the spatial pattern of pregnant women immune to Rubella (CMV) was similar to the pattern presented by non-immune pregnant women, as presented in Yue and Lee [10].

### Stastical analysis

Excel software and analysed using Statistical Package for the Social Sciences (SPSS) version 20. The exploratory analysis of the categorical variables and quantitative variables are presented as percentages (± SD). Differences between subgroups were assessed by Chi-square analysis and p-values are presented. Bivariate and multivariate logistic regression to assess the effect of different risk factors on rubella virus seroprevalence. The level of statistical significance was set as p < 0.05.

## Results

The distribution of the serostatus of the 396 pregnant women is summarized in Table 1. Among them, 382 (96.5%) had anti-CMV IgG antibodies, 8 (2.0%) had anti-CMV IgG and IgM antibodies and 6 (1.5%) were seronegative. For Rubella virus, 347 (87.6%) were positive for anti-IgG, 4 (1.0%) positive for anti-IgG and IgM, and 45 (11.4%) were seronegative. The majority (n = 335; 84.6%) were immune to both CMV and RV infections, while 8 (2.0%) had active CMV infection and 4 (1.0%) active RV infection but none had an active dual infection. Five women (1.2%) were susceptible to only CMV infection, 43 (10.9%) to only RV infection, and 1 (0.3) to both infections. None had a recent infection with CMV or RV.

**Table 1:** The serostatus of CMV and Rubella infection of 396 pregnant women from Luanda (Angola)

	CMV			Total n (%)
	Previous infection or immune n (%)	Active (primary/latent) infection n (%)	Susceptible to primary infection n (%)	
Rubella				
Previous infection or immune	335 (84.6)	7 (1.7)	5 (1.2)	347 (87.6)
Active (primary/latent) infection	4 (1.0)	0	0	4 (1)
Susceptible to primary infection	43 (10.9)	1 (0.3)	1 (0.3)	45 (11.4)
Total n (%)	382 (96.5)	8 (2)	6 (1.5)	396 (100)

The age range of the women was 15 to 47 years while the mean age ± SD was 28.4 ± 6.2 years; more than half (66.2%) were 26 to 47 of age (Table 2). Regarding educational level, 152 (38.4%) had low education (3 were illiterate and 149 basic education) and 244 (61.6%) had high school or higher education (200 high school education and 44 higher education). The majority lived in the municipalities of Belas, Cacuaco, Viana and Cazenca (216; 54.5%) and 180 (45.5%) lived in the municipality of Luanda. Regarding employment, while 63 (15.9%) were homemakers the majority women worked outside home (333; 84.1%): in public administration services (150; 37.9%), street vendors (49; 12.4%), store employees (39; 9.8%), restaurant waitress (31; 7.8%) and 64 (16.2%) were students. The majority was single (68.2%), had more than one children (63.9%) and reported had pre-natal consultation (97.7%). Among the pregnant women, 173 (43.7%) had a history of abortion and 27 (6.8%) had spontaneous abortion. About two-thirds of the participants (64.4%) reported having basic sanitation at home while 141 (35.6%) did not have (Table 2).

**Table 2:** Sociodemographic characteristics and serostatus of CMV and Rubella-infection of pregnant woman in Luanda province, Angola

Variable	Previous infection or immune n (%)	Active infection n (%)	Susceptible to infection n (%)	Total n (%)	p-value^&^
Age					
≤ 25 years old	112 (33.5)	3 (25.0)	19 (38.8)	134 (33.8)	0.5192
26–47 years old	223 (66.5)	9 (75.0)	30 (61.2)	262 (66.2)	
Education					
Low (up to elementary school)	134 (40.0)	4 (33.4)	14 (28.6)	152 (38.4)	0.157
High school or higher education)	201 (60.0)	8 (66.6)	35 (71.4)	244 (61.6)	
Residence					
Belas, Cacuaco, Viana, Cazenga	181 (54.0)	7 (58.3)	28 (57.1)	216 (54.5)	0.759
Luanda	154 (46.0)	5 (41.7)	21 (42.9)	180 (45.5)	
Employment					
Public administration, street vendor, saleslady restaurant waitress or student	280 (83.6)	11 (91.7)	42 (85.7)	333 (84.1)	0.836
Homemakers	55 (16.4)	1 (8.30)	7 (14.3)	63 (15.9)	
Marital status					
Single	230 (68.7)	8 (66.6)	32 (65.3)17	270 (68.2)	0.626
Married	105 (31.3)	4 (33.4)	(34.7)	126 (31.8)	
Gestational age					
1st trimester	172 (51.3)	8 (66.6)	22 (44.9)	202 (51)	0.166
2nd and 3rd trimestre	163 8 (48.7)	4 (33.4)	27 (55.1)	194 (49)	
Number of births					
0	67 (20.0)	2 (16.6)	17 (34.7)	86 (21.7)	0.026*
≥ 1	268 (80.0)	10 (83.4)	32 (65.3)	310 (78.3)	
Children at home					
0 or 1	115 (34.3)	5 (41.7)	23 (46.9)	143 (36.1)	0.110
2 or more	220 (65.7)	7 (58.3)	26 (53.1)	253 (63.9)	
Spontaneous abortion					
Yes	12 (3.6)	9 (75.0)	6 (12.2)	27 (6.8)	0.017*
No	323 (96.4)	3 (25.0)	43 (87.8)	369 (93.2)	
History of miscarriages					
Yes	150 (44.8)	8 (66.6)	15 (30.6)	173 (43.7)	0.065
No	185 (55.2)	4 (33.4)	34 (69.4)	223 (56.3)	
Hepatitis B					
Positive	109 (32.5)	5 (41.7)	13 (26.5)	127 (32.1)	0.511
Negative	226 (67.5)	7 (58.3)	36 (73.5)	269 (67.9)	
HIV status					
Positive	55 (16.4)	1 (8.3)	2 (4.1)	58 (14.6)	0.018*
Negative	280 (83.6)	11 (91.7)	47 (95.9)	338 (85.4)	
Pre-natal consultation					
Yes	327 (97.6)	11 (91.6)	49 (100)	387 (97.7)	0.603
No	8 (2.4)	1 (8.4)	0 (00.0)	9 (2.3)	
Access to basic sanitation					
Yes	214 (63.9)	9 (75.0)	32 (65.3)	255 (64.4)	0.875
No	121 (36.1)	3 (25.0)	17 (34.7)	141 (35.6)	
Total	335	12	49	396	

^&^p values of the Chi square test between previous + active infection and susceptible; *p < 0.05

In relation to the gestation age, the frequency of CMV and Rubella infection (previous infection or immune and active infection) was higher among pregnant women in the first trimester (51.3% and 66.6%) followed by pregnant women in the second and third trimester (48.7% and 33.4%) (Table 2). Regarding the the parity (number of births), the frequency of previous infections or immune and active infection was higher in pregnant women with 1 birth or more (80% and 83%) followed by women with 0 birth (20.0% and 16.6%) (Table 2).

We also studied the frequency of CMV and Rubella infection in pregnant women with hepatitis B and human immunodeficiency virus (HIV) infections (Table 2). In relation to Hepatitis B, 127 (32.1%) pregnant women presented a positive result, of which 109 had a previous or immune infection to CMV and Rubella and 5 a active infection. For the HIV, 58 (14.6%) pregnant women presented a positive result, of which 55 had a previous infection or immune to CMV and Rubella and 1 a active infection (Table 2).

Significant differences were found in the serostatus of CMV and Rubella-infection between women having zero or more than one birth (p = 0.026), reporting (or not) spontaneous abortion (p = 0.017) and having (or not) HIV positive (p = 0.018) (Table 2).

The seroprevalence of Rubella infection in Angolan pregnant women according to independent categorical variables evaluated in this study are summarized in Table 3. In the bivariate logistic regression analysis, the variables of number of births (OR 2.478; CI 1.144–5.374), history of miscarriages (OR 2.062; CI 1.069–4.194), and spontaneous abortions occurred during the study (OR 3.048; CI 1.135–7.394), were predictors of RV infection among pregnant women (Table 3). Other factors such as maternal age, gestacional age, residence, occupation, educational status, access to basic sanitation, hepatitis B, were not associated with seropositivity. The multivariate logistic regression analysis (adjusted to age) confirm a significant increased risk of rubella in women without children (OR 2.673; CI 1.026–7.007) and who had spontaneous abortion (OR 3.232; CI 1.192–7.952). The women positive to HIV had highlook probability to have seropositivity to Rubella (OR 4.121; CI 1.217–25.748), however not statistically significant (p 0.055). For CMV, the statistical analysis of the risk factors associated with the infection not produced valid information due the small number of CMV-seronegative women.

**Table 3:** Binomial logistic regression models for the final analysis of risk factors associate for seropositivity of IgG anti-rubella antibodies in 396 pregnant woman in Luanda province, Angola

Variable	OR (95% IC)	p-value	OR (95% IC)	p-value
	Unadjusted		Adjusted by age	
Age				
≤ 25 years old (ref)				
25–29 years old	0.962 (0.439–2.059)	0.923		
≥ 30 years old	0.609 (0.286–1.272)	0.190		
Residence				
Belas	1.226 (0.540–2.667)	0.613	1.214 (0.533–2.649)	0.631
Cacuaco	0.651 (0.034–3.563)	0.688	0.665 (0.035–3.702)	0.704
Viana	1.242 (0.547–2.704)	0.590	1.279 (0.561–2.796)	0.543
Cazenga	0.977 (0.218–3.131)	0.973	1.020 (0.227–3.289)	0.976
Luanda (ref)				
Education				
Low (up to elementary school)	0.619 (0.304–1.196)	0.167	0.615 (0.300–1.197)	0.166
High (high school or higher education) (ref)				
Employment				
Public administration	1.510 (0.704–3.395)	0.299	1.761 (0.792–4.121)	0.175
Homemakers	1.033 (0.340–2.863)	0.951	1.102 (0.361–3.079)	0.856
Student	1.402 (0.516–3.664)	0.492	1.064 (0.368–3.001)	0.905
Street vendor, saleslady and restaurant waitress (ref)				
Marital status				
Single (ref)				
Married	1.347 (0.696–2.543)	0.363	1.390 (0.715–2.639)	0.319
Gestational age				
1st trimester (ref)				
2nd and 3rd trimester	1.346 (0.722–2.536)	0.351	1.377 (0.737–2.604)	0.317
Number of births				
0	2.478 (1.144–5.374)	0.0203*	2.673 (1.026–7.007)	0.0439*
1	1.692 (0.789–3.627)	0.1724	1.694 (0.748–3.825)	0.2020
2 or 3 (ref)				
Children at home				
0 or 1	1.818 (0.969–3.404)	0.0606	1.689 (0.840–3.394)	0.139
2 or more (ref)				
Spontaneous abortion				
Yes	3.048 (1.135–7.394)	0.018*	3.232 (1.192–7.952)	0.0139*
No (ref)				
History of miscarriages				
Yes (ref)				
No	2.062 (1.069–4.194)	0.0364*	2.048 (0.957–4.508)	0.0676
Hepatitis B				
Positive	0.844 (0.413–1.636)	0.627	0.839 (0.410–1.629)	0.617
Negative (ref)				
HIV				
Positive	4.168 (1.235–26.00)	0.053	4.121 (1.217–25.748)	0.0553
Negative (ref)				
Access to basic sanitation				
Yes (ref)				
No	0.892 (0.452–1.697)	0.735	0.867 (0.437–1.653)	0.672

*OR* Odds ratio, *CI* confidence interval

*Statistically significant (p < 0.05)

**Fig. 1:**
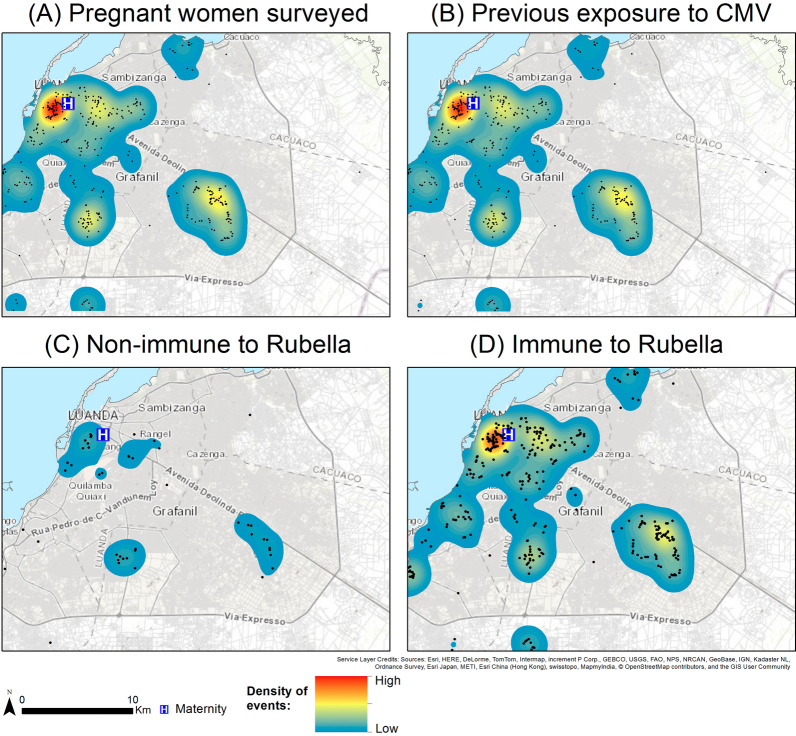
Geographical distribution and Gaussian kernel density surface map of pregnant women (**A**) with anti-CMV antibodies (**B**), non-immune to Rubella virus (**C**) and immune to Rubella virus (**D**) in Luanda, Angola

There was a high density of CMV and Rubella infections among women whose residence was near Lucreta Paim Maternity in Luanda (Fig. 1). The results of Ripley’s K-function analysis showed that the geographical distributions of pregnant women with and without antibodies to CMV and Rubella were similar, and clustered around each other.

## Discussion

The present study was performed to investigate the seroprevalence of CMV and rubella infections among pregnant women attending for antenatal care in the northern Angolan city of Luanda. We found an overall seroprevalence of CMV infection of 98.5% and Rubella infection of 88.6%. The majority (84.6%) had previous dual infection. This is the first study of this nature.

Concerning CMV seroprevalence in pregnant´s women, studies in the African continent showed high rates in East Africa (72–89%) and even higher North, Southern and West (100%) [11]. The seroprevalence of CMV among our patients is similar to that reported from other developing countries, both African [12–20] and non-African [19–24] but higher than the prevalence in developed countries [25–32]. The lower levels of education and socioeconomic status as well as the higher prevalence of poor hygienic conditions in developing countries could be factors associated with the higher prevalence of CMV in these countries [33–35].

Pregnant women with pre-existing anti-CMV antibodies, remain at risk of CMV reactivation or reinfection and vertical transmission to the fetus [36]. So, the high IgG seropositivity (previous infection) in womens from Luanda may not be reassuring.

An additional finding of our study was that the majority of pregnant women were in the first trimester of gestation, the period of highest risk vertical transmission [37]. CMV stands out as major cause of congenital infection, reaching rates between 0.2 and 2.6% of the total number of births worldwide, being responsible for cases of neonatal mortality and morbidity [38]. Fetal CMV infection occurs in approximately 40% of cases of maternal primary infection [39]. Therefore, it would be beneficial to inform pregnant women about the need for follow-up to detect prenatal infection and to plan appropriate intervention such as the use of drugs to control infection and/or prevent infection [16].

The seroprevalence of rubella among pregnant women in the present study (88.6%) was similar than reported from other African countries [40–44] as well from countries in America, Europe and the Middle East [45–48]. In contrast, the seroprevalence in this study is higher than reports from Democratic Republic of Congo, Sudan and Nigeria (50–68%) [49–51]. These variations might be due to the endemicity of the rubella virus, the sample and the laboratory methods used, and the presence or absence of rubella vaccination in their immunization programs.

Despite overall the high seroprevalence of rubella infection, 11.4% of the pregnant woman were seronegative (susceptible to infection). The babies of these women could be ar risk of CRS [40, 52]. Attention should be paid to such women in order to reduce the risk of CRS in their future pregnancies.

Our results showed that 87.6% of the pregnant women had IgG levels of > 10 IU/mL (immune). None had a previous history of rubella vaccination. This might be due to the prenatally, as rubella infection is common among children and teenagers in some countries [40, 53].

In the present study, 1% of women had both rubella IgM and IgG antibodies (active infection) and were in the first and second trimester of pregnancy. In multivariate analysis, a significant association was found between rubella IgG positivity and spontaneous abortions. This is in agreement with reports of rubella as causefor miscarriages in many countries [46, 54]. Therefore, more attention should given to pregnant women with recent or acute infections.

There was no significant relationship between anti-Rubella antibodies positivity and socio-demographic characteristics. Similar findings have been reported from Namibia [42], Southern Ethiopia [55], and Nigeria [56]. All the participants in our study were resident in urban areas. The high population density in these areas might be associated with an increased risk of RV infection [40], as was reported in the pre-vaccine era in other countries [57–59].

In the present study, significant differences were found in the serostatus of CMV and Rubella-infection between women having (or not) HIV positive (p = 0.018): 58 (14.9%) pregnant women had HIV positive results, of which 55 presented positive results to CMV and Rubella infection. WHO recommends that all pregnant women should be tested for HIV at the first prenatal visit. There is a need to improve prenatal services in our setting to ensure that all women are counseled and tested for HIV (11).

Vaccination is essential to reduce the circulation of the Rubella virus, and prevent congenital rubella which could be associated with permanent sequelae including glaucoma, cataract, cardiac malformation, delayed growth, deafness and others. Therefore, prevention should be focused [60], and the WHO has recommended vaccination as a strategy for reducing the transmission of rubella virus infection [52]. Vaccine was included in the Angolan national vaccination plan in April 2018 although the initial stage only covered children up to 14 years of age [61].

Our study had some limitations regarding the small number of CMV-seronegative women that did not allow for analysis of the risk factors associated with CMV infection. It will be important address these limitation in future studies.

## Conclusion

Overall, this study showed that while there is a high prevalence of previous or active CMV and/or rubella infection among pregnant women in Luanda, a small but important proportion of about 11% are susceptible to rubella virus infection. Rubella vaccination should be offered to these women.

## Supplementary Information

**Additional file 1.** Ethics Committee, República de Angola, Ministério da Saúde.

**Additional file 2.** Questionnaire/Questionário de Recrutamento.

## Data Availability

The datasets used and analysed during the current study are available from the corresponding author on reasonable request.

## Abbreviations

95% CI: 95% Confidence intervals
COI: Cut-off índex
CRS: Congenital Rubella Syndrome
ECL: Electrochemiluminescence
HIV: Human immunodeficiency virus
LPMH: Lucrécia Paim Maternity Hospital
MDI: Material deprivation index
NCCLS: National Committee for Clinical Laboratory Standards
OR: Odds ratio
SPSS: Statistical Package for the Social Sciences
TORCH: Toxoplasmosis, Other (syphilis, varicella-zoster, parvovirus B19), Rubella, Cytomegalovirus and Herpes
UNESCO: United Nations Educational, Scientific and Cultural Organization
WHO: World Health Organization
