# Supplementary material for: Seroepidemiology study of Cytomegalovirus and Rubella in pregnant women in Luanda, Angola: geospatial distribution and its association with socio-demographic and clinical-obstetric determinants
Source: BMC Infect Dis. 2022 Feb 5;22:124. doi: 10.1186/s12879-022-07087-x (PMC8818202; doi:10.1186/s12879-022-07087-x)
Supplement: Supplementary file 2 — Additional file 2. Questionnaire/Questionário de Recrutamento. [file 12879_2022_7087_MOESM2_ESM.pdf]

QUESTIONNAIRE

Date ___/___/___

Internal Number:__________

Laboratory: _____________________________

Process number: ___________________

Personal data

Residence: ___________________

Level of education: __________

Age: _______________ Gestational Age: ____________

Number of births

0 ☐

1 and 2 ☐

≥3 ☐

Did you do prenatal consultation in all pregnancies?

No ☐

Yes ☐

Risk factors

1. In the residence, do you have a basic sanitation? (water treated, collection refuse, sewage system):

Yes ☐

No ☐ If not, which one do not have?

Water treated ☐

Collection refuse ☐

Sewage system ☐

2. Do you currently have pets at home?

No ☐

Yes ☐ If so, which? Cats ☐

Dogs ☐

Others ☐

3. Currently has contact with animals other than yours at home the of relatives or other households.?

No ☐

Yes ☐ If so, which?

Cats ☐

Dogs ☐

Others ☐

4. Do you have contact with cats other than yours?

No ☐

Yes ☐ If so, how often?

Very frequent ☐

Less frequent ☐

Rarely ☐

5. In the case you have pet cats, where do they defecate?

Inside home ☐

In the vicinity of the house ☐

Far from the house ☐

6. If you have pet cats, **what is your main food?**

Ration ☐

Leftover food (cooked) ☐

Restos de carne crua ☐

7. Do you know of rodents at or near your home?

No ☐

Yes ☐

8. Do you have a garden in your residence?

No ☐

Yes ☐ In the case of yes this is fenced, preventing the entry of cats

No ☐

☐

Yes

9. Do you carry out soil-related activities (gardening, agriculture, etc.)?

No ☐
Yes ☐

10. Do you have farmed animals for your own consumption?

No ☐
Yes ☐ if yes, which one

Cattle ☐
Pigs ☐
Birds ☐
Others ☐

11. Do you have habits of consuming meat from animals slaughtered in hunting such as birds, rabbits, wild boars etc.

No ☐
Yes ☐

12. Do you eat raw or undercooked meat?

No ☐
Yes ☐ if yes, from which animals?

Cattle ☐
Pigs ☐
Birds ☐
Others ☐

if yes, how often?

Very common ☐
Less common ☐
Rarely ☐

13. Do you consume unpasteurized milk or dairy products?

No ☐

Yes ☐

if yes, how often?

Very common ☐

Less common ☐

Rarely ☐

14. Do you always wash fruits and vegetables before consuming them?

No ☐

Yes ☐

if yes, how often?

Very common ☐

Less common ☐

Rarely ☐

15. Do you eat raw or undercooked eggs?

No ☐

Yes ☐

if yes, how often?

Very common ☐

Less common ☐

Rarely ☐

16. Do you consume water from well / borehole?

No ☐

Yes ☐ if yes, how often?

Very common ☐

Less common ☐

Rarely ☐

17. Have you recently had a blood transfusion?

No ☐

Yes ☐

18. Recently had you a needle stick / syringe accident?

No ☐

Yes ☐

Questionário

Data ___/___/___

Número interno:_________

Laboratório: __________________________

Número do Processo: _________________

Dados Pessoais

Residência: _________________

Nível de escolaridade: _________

Idade: ______________Idade Gestacional: ___________

Quantas vezes já esteve grávida?

Uma ☐

Duas ☐

Mais ☐

Fez consulta pre - natal em todas as gestações?

Não ☐

Sim ☐

Factores de risco

1. Na residência, possui saneamento básico? (água tratada, colheita de lixo, sistema de esgoto):

Sim ☐

Não ☐ Se não, quais não possui?

Água ☐

Esgoto ☐

Lixo ☐

2. Tem animais de estimação actualmente em casa?

Não ☐

Sim ☐ Se sim, qual? Gatos ☐

Cães ☐

Outros ☐

Qual é o número destes animais no domicílio? __________.

Os animais têm acesso ao interior de sua residência?

Não ☐

Sim ☐

3. Atualmente tem contacto, com frequência, com animais, que não os seus, em casa de parentes ou outras residências?

Não ☐

Sim ☐ Se sim, quais? Gatos ☐

Cães ☐

Outros ☐

4. Tem contacto com outros gatos que não sejam os seus?

Não ☐

Sim ☐ Se sim com que frequência?

Muito frequente ☐

Pouco frequente ☐

Raramente ☐

5. Caso tenha gatos de estimação, onde é que eles defecam?

Dentro da casa ☐

Na proximidade da casa ☐

Distante da casa ☐

6. Caso tenha gatos de estimação, qual é o seu principal alimento?

Ração ☐

Restos de comida (cozinhada) ☐

Restos de carne crua ☐

7. Sabe da existência de roedores no seu domicílio ou nas proximidades?

Não ☐

Sim ☐

8. Tem horta na sua residência?

Não ☐

Sim ☐ Se sim esta é cercada, evitando a entrada de gatos?

Não ☐

Sim ☐

9. Realiza atividades ligadas ao solo (jardinagem, agricultura, etc.)?

Não ☐

Sim ☐

10. Tem animais de criação para consumo próprio?

Não ☐

Sim ☐ Se sim, quais?

Gado ☐

Porcos ☐

Aves ☐

Outros ☐

11. Tem hábitos de consumir carne proveniente de animais abatidos em caça como pássaros, coelhos, javalis etc.

Não ☐

Sim ☐

12. Consome carne crua ou mal cozida?

Não ☐

Sim ☐ Se sim, de que animais? Gado ☐

Porco ☐

Ave ☐

Outros ☐

Se sim, com que frequência?

Muito frequente ☐

Pouco frequente ☐

Raramente ☐

13. Consome leite ou lacticínios não pasteurizados?

Não ☐

Sim ☐ Se sim, com que frequência?

Muito frequente ☐

Pouco frequente ☐

Raramente ☐

14. Lava frutas e verduras sempre antes de consumi-las?

Não ☐

Sim ☐ Se sim, com que frequência?

Muito frequente ☐

Pouco frequente ☐

Raramente ☐

15. Consome ovo cru ou mal cozido?

Não ☐

Sim ☐ Se sim, com que frequência?

Muito frequente ☐

Pouco frequente ☐

Raramente ☐

16. Consome água de poço/furo?

Não ☐

Sim ☐ Se sim, com que frequência?

Muito frequente ☐

Pouco frequente ☐

Raramente ☐

17. Recentemente fez alguma transfusão de sangue?

Não ☐

Sim ☐

18. Recentemente teve alguma picada/acidente de agulha/seringa?

Não ☐

Sim ☐
